# Comparison of the inward leakage rate between N95 filtering facepiece respirators and modified surgical masks during the COVID-19 pandemic

**DOI:** 10.1265/ehpm.23-00303

**Published:** 2024-02-16

**Authors:** Kazunari Onishi, Masanori Nojima

**Affiliations:** 1 Division of Environmental Health, Graduate School of Public Health, St. Luke’s International University, 3-6-2 Tsukiji Chuo-ku, Tokyo 104-0045, Japan; 2 Center for Translational Research, The Institute of Medical Science Hospital, The University of Tokyo, 4-6-1 Shirokanedai, Minato-ku, Tokyo 108-8639, Japan

**Keywords:** Infection prevention, Mask, Personal protective equipment, Fit test, Respirators, Inward leakage rate

## Abstract

**Background:**

Owing to shortage of surgical and N95 filtering facepiece respirators (FFRs) during the COVID-2019 pandemic, various masks were developed to prevent infection. This study aimed to examine the inward leakage rate (ILR) of sealed face masks and modified surgical masks using a quantitative fit test and compared it with the ILR of unmodified N95 FFRs.

**Methods:**

We conducted paired comparisons of ILRs of bent nose-fit wire masks, double masks, and N95 FFRs from October to December 2021. To measure the protective effectiveness of masks, participants wore masks, and the number of particles outside and inside the mask were measured. The ILR was based on the percentage of particles entering the mask using a fit tester.

**Results:**

We enrolled 54 participants (20 men and 34 women) in this study. The median ILR for surgical masks without and with a W-shaped bend in the nose-fit wire were 96.44% and 50.82%, respectively. The nose-fit wire adjustment reduced the ILR of surgical masks by a mean of 28.57%, which was significantly lower than the ILR without adjustment (*P* < 0.001). For double masks, with surgical or polyurethane masks on top of the W-shaped mask, the ILR did not differ significantly from that of N95. Although the filtration performance of double surgical masks matched that of N95 masks, their ILR was notably higher, indicating that double masks do not provide equivalent protection.

**Conclusions:**

Wearing N95 masks alone is effective in many cases. However, surgical mask modifications do not guarantee consistent effectiveness. Properly selected, sealed masks with a good fit overcome leakage, emphasizing their crucial role. Without evidence, mask-wearing may lead to unexpected infections. Education based on quantitative data is crucial for preventing adverse outcomes.

## Graphical Abstract

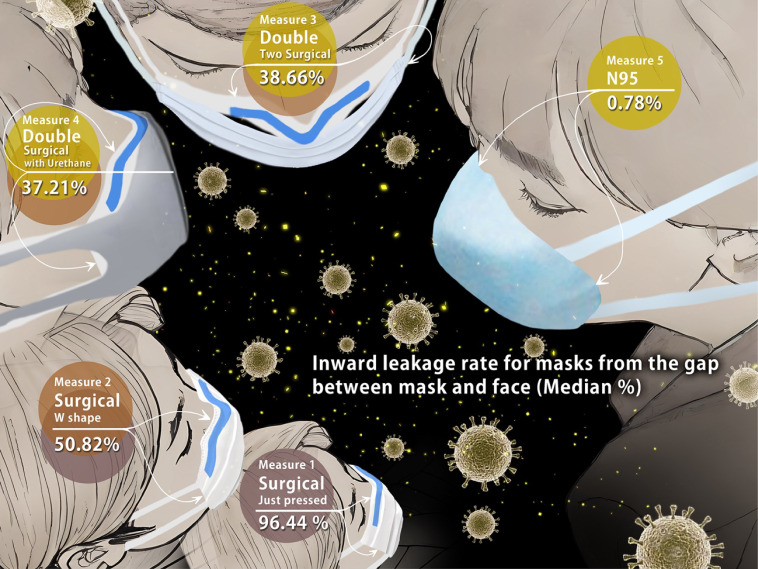

## Background

The severe acute respiratory syndrome coronavirus 2 (SARS-CoV-2), the cause of coronavirus disease 2019 (COVID-19), has infected 24 million people in Japan (as of May 9, 2023) and resulted in 74694 deaths. SARS-CoV-2 is a transmissible virus that infects the upper and lower respiratory tracts [1]. Concerns about the effectiveness of masks have spread worldwide. Regardless of SARS-CoV-2 infection, the public health management strategy to mitigate most infections is universal masking [2]. A correctly worn mask that completely covers the nose and mouth can help reduce virus transmission.

SARS-CoV-2 continues to mutate, with new cases of infection caused by the Omicron (B.1.1.529) variant being reported globally [3]. Since the start of the pandemic, Japan’s adherence to universal masking has been cited by media and government reports as a reason for the low number of positive cases. Yet, by July 2022, Japan had the highest number of cases worldwide [4]. This might be attributed to the transmission mode, which is not limited to droplets and direct contact but also involves airborne routes [5, 6], indicating the need for strict mask-wearing techniques to prevent aerosol droplet infiltration and infection.

Sealed N95 filtering facepiece respirator (FFR) filters were tested at the US National Institute for Occupational Safety and Health (NIOSH) laboratories, and they demonstrated a particle filtration efficiency (PFE) of at least 95% (0.075 ± 0.02 µm) by 85 L/min [7]. In Japan, disposable masks worn to prevent the transmission of solid particles, with a DS2 certification (a Japan equivalent of the US N95 standard), demonstrated a PFE of at least 95% (0.06–0.1 µm) by 40 L/min, according to tests by the Ministry of Health, Labor, and Welfare. The PFE of another type of disposable unwoven surgical face mask, evaluated using the American Society for Testing and Materials F 1215 (ASTM F 1215) standard test method, was found to be ≥95% for aerosols at a face velocity of 0.5–25 cm/s using 0.1 µm polystyrene latex particles [7].

In Japan, companies conduct independent PFE tests using the ASTM methods. JIS T 9001 medical face masks were developed in 2021 by the Japanese Standards Association and were tested formally using the ASTM guidelines at a face velocity of 6.5 cm/s, demonstrating a PFE of >95% using 0.1 µm polystyrene latex particles. Masks with filters passing this test can be assumed to prevent infiltration of particles at the regular respiratory flow rates in humans [8]. These tests do not include the effects of unfiltered air leakage through the gaps in face seals. Although the fit factor (FF) or inward leakage rate (ILR) test results for the N95 and DS2 masks are submitted as reference data, they are not required for certification. However, it is important to also consider leakage through the gaps when ascertaining the effectiveness of the mask [9–12].

Due to shortage of surgical masks, items such as kitchen paper, buffs, and gauze masks without guaranteed filter protection have also been used for airborne infection control [13, 14]. Even when the supplies of surgical masks and N95 FFRs returned to normal, modifications of the mask-wearing method to maximize overall mask performance were attempted, such as improving the efficiency of the filter or creating an adequate fit for the face [9, 13, 15–17]. Other modifications included fastening the string of the mask with a hook at the back, tying the mask behind the neck and head with either elastic bands or ties, or wearing two masks (double mask). The double mask (surgical mask underneath and cloth mask on top) technique has been tested using a simulated cough with exposure of the receiver and was found to prevent the infiltration of particles effectively [18, 19].

Additionally, droplet behavior simulations have been used to test the effectiveness of improving the fit, performance, and type of mask [20–22]. However, these laboratory-based experiments and simulations cannot be generalized and interpreted as representing the effectiveness of these masks in real-world settings.

Mask fit can be measured using quantitative fit testing (QNFT) [10, 23]. A fit test is a method of gauging the best fit of a mask for a person’s face [24]. Fit tests are designed to be performed with N95 FFRs and highly functional masks such as powered air-purifying respirators (PAPR) by pre-selecting a mask to fit the face. A fit check (seal check) ensures that there are no leaks immediately before entering a contaminated space without damaging the mask.

QNFT is primarily conducted using a fit tester utilizing condensation nucleus counter (CNC) technology (particle size 0.02–1 µm) following the fit testing protocol approved by the US Occupational Safety and Health Administration (OSHA) as a regulatory standard. On the other hand, Optical particle counting (OPC) based fit tester (particle size ≥0.3 µm) is commonly used for fit checks. OPC testers were discussed as not being a substitute for CNC testers, but the validation of both has been reported [25, 26].

Surgical masks do not have standardized fit testing and fit checking. Therefore, to examine the effectiveness of modified surgical masks and the validity of the formal fit test method, we performed a fitting check and evaluation of surgical masks under specified conditions, while the mask was being worn by the participants.

We focused on prevention of infection caused via airborne transmission of microdroplets using various mask types. This study aimed to determine the inward leakage of a face sealed modified surgical mask using QNFT and to compare the performance of these masks with that of unmodified surgical masks and N95 FFRs.

## Methods

### Study participants

The study population included volunteers from the general population and healthcare workers (HCWs), including nursing students, who were tested between October and December 2021 before the sixth wave of the COVID-19 epidemic in Japan. The study was conducted at a university hospital in Tokyo. Participants were not allowed to eat, drink, or smoke immediately before the measurement because this might affect the number of particles in the mask.

### Measurement of face size

**Fig. 1:**
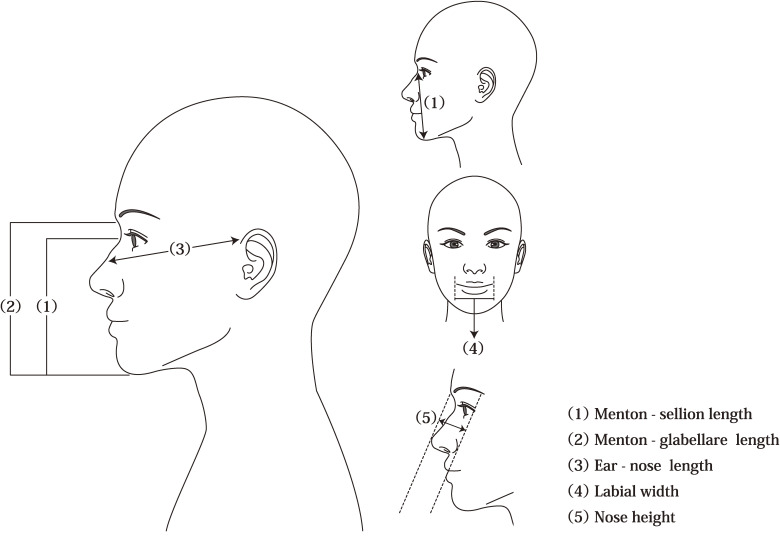
Measurement criteria for face size

The following five face-size parameters were measured: (1) menton-sellion length (MSL), the concave part of the base of the nose; (2) menton-glabella length; (3) ear-nose length, the distance from the top of the ear to the top of the nose (the ear is the part of the surgical mask where the rubber string touches and the nose is the part of the nose-fit wire); (4) labial width (LW), the distance between the left and right angles of the mouth; and (5) nose height (NH), measured vertically to the height of the nose, making the face appear flat (Fig. 1). MSL and LW were measured according to the NIOSH manual. We included the menton-glabella length, as finding the sellion in Asian faces is sometimes difficult. Ear-nose length is frequently used to guide the selection of surgical mask sizes in Japan. In this study, NH was measured to determine nasal leakage.

### Masks and respirators used in this study

We used the following four types of masks (Table 1). Mask A was the mask worn by the participant at the time of recruitment. We prepared Masks B, C, and D. Surgical Mask B was made of an unwoven fibrous material without sterilization (procedure mask) that passed the PFE test in ASTMF 2100-19 and JIS T 9001 and reduced fine particulate matter (FPM) by >99%. Mask C was a foam sponge urethane mask, which is sometimes used as a substitute for regular hygiene masks in Japan because of its good breathability and comfortable fit. During the COVID-19 pandemic, Mask C was often used in combination with a surgical mask (Mask B) as a double mask. Mask D was the N95 FFR, a cup-type dust mask with a round-contact face cushion (certified according to both N95 and DS2 criteria). Mask D is the most widely used model in Japanese hospitals. The value of Measure 0 was excluded for participants wearing the N95 FFR. We prepared both regular and small sizes of Masks B and C. To avoid significant issues with face fit, a small size (ear-nose length <12 cm) was chosen for participants with smaller faces.

**Table 1:** Physical properties of the masks used in this study

	**Mask A^a^**	**Mask B**	**Mask C**	**Mask D**
		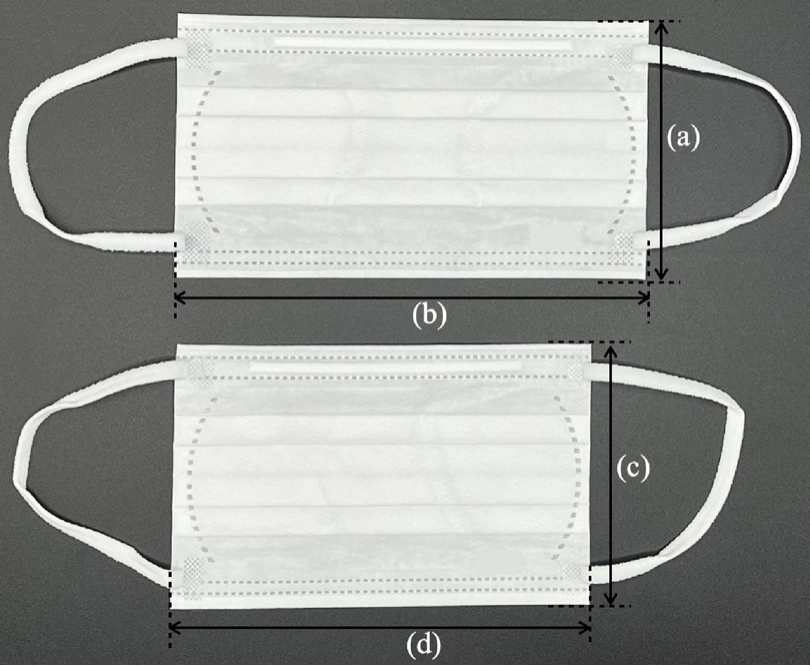	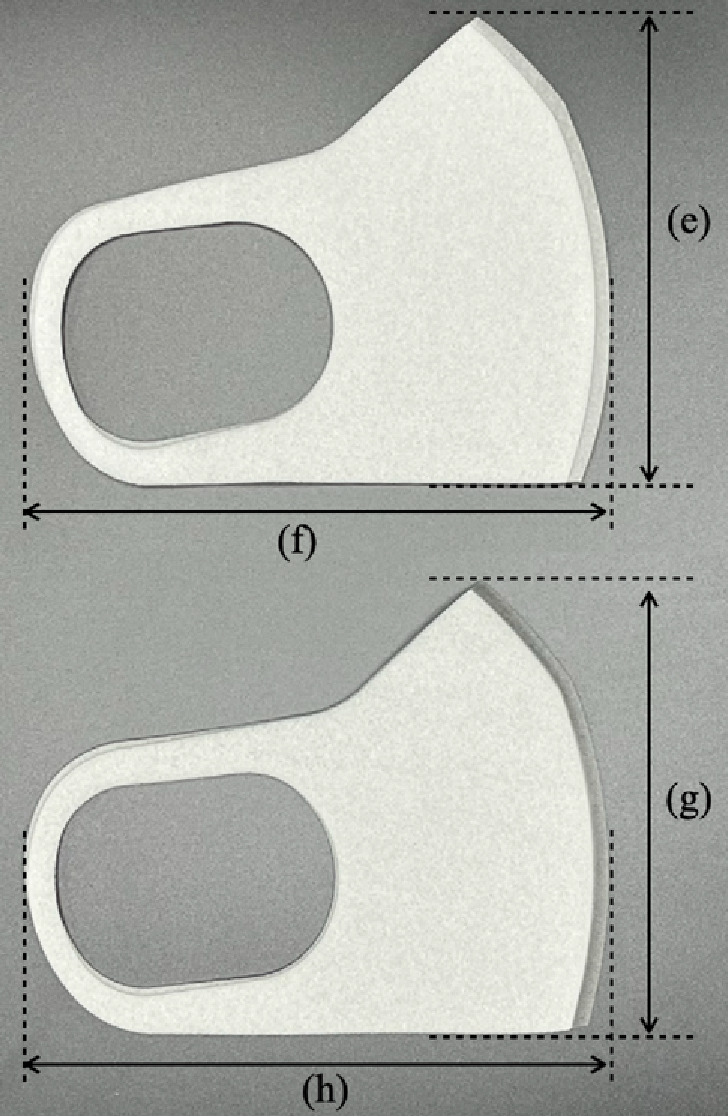	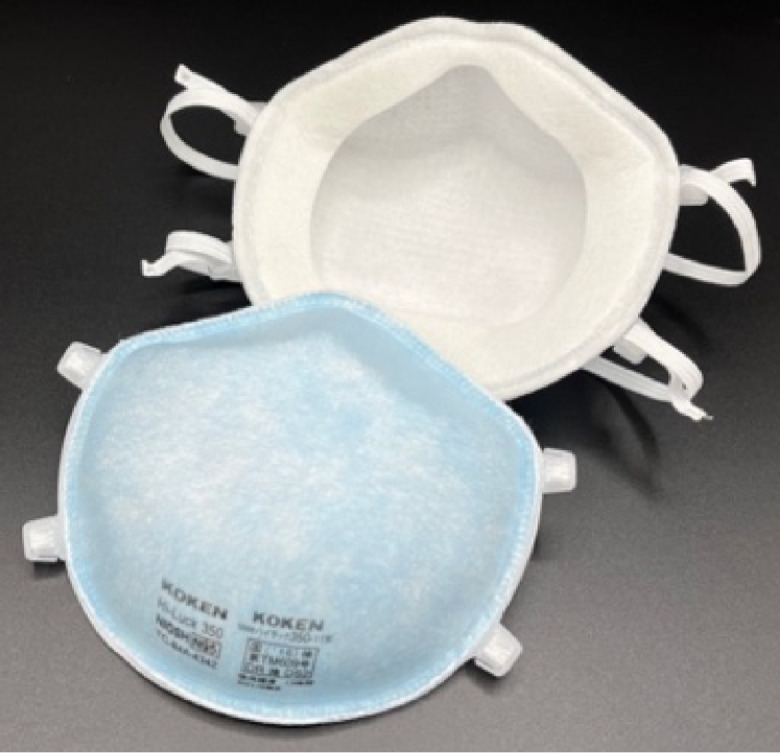
**Name**	NA	Surgical mask (without sterilization)	Urethane mask	Filtering facepiece respirators (FFRs), dust mask
**Shape**	NA	Flat and flexible with 4 pleats	Flat without pleats	Rigid cup-type
**Material**	NA	3 layers of polyethylene filter (melt blown non-woven fabrics)	Polyurethane foam	5 layers of filter (polyester, polypropylene)
**Size^b^**	NA	Regular (a) 91.84 ± 0.20 (b) 165.08 ± 0.62 Small (c) 91.27 ± 0.35 (d) 146.28 ± 0.48	Regular (e) 125.95 ± 0.67 (f) 157.13 ± 0.89 Small (g) 118.95 ± 0.19 (h) 154.94 ± 0.71	Standard (one size only)
**National standard ** **approved**	NA	No	No	N95 (NIOSH), DS2 (MHLW)
**Filter-collection ** **efficiency (85 L/min)**	NA	(Single) 94.3% (Double) 99.3%	8.5%	96.7%
**Inhalation resistance ** **(85 L/min)**	NA	(Single) 53 Pa (Double) 102 Pa	4 Pa	90 Pa
**Other features**	Urethane mask N = 1 Cloth mask N = 1 Cloth with unwoven filter N = 2 Surgical mask N = 37 KN95 N = 3 KF94 N = 3 N95 (same as Mask D) N = 7	PFE test (ASTM) >99.0%, Ear hook part is elastic band	Tested to cut >30 µm PM >99.0%, The material of the ear hook is also made of urethane	Braided headband, soft inner material adhere to the face
**Used in^c^**	Measure 0	Measures 1, 2, 3, 4, 6, 7	Measure 4	Measures 5, 8

ASTM, American Society for Testing and Materials International; MHLW, Ministry of Health, Labor, and Welfare; NA, not applicable; NIOSH, National Institute for Occupational Safety and Health; PFE, particle filtration efficiency; PM, particulate matter.

^a^ Mask worn by the participant at recruitment.

^b^ Ten specimens were tested for each mask.

^c^ Measure 0: Mask A, the mask that the participant was wearing when recruited; Measures 1 and 6 (Surgical): Mask B, worn with the pleats spread out and the filter wire on the nose pressed down; Measures 2 and 7 (Surgical-W): Mask B with the nose nose-fit wire adjusted to a W shape, according to the shape of the participant’s nose, prior to wearing; Measure 3 (S-S double): A double mask with two layers of Mask B; Measure 4 (S-U double): A double mask with Mask C worn over Mask B; Measures 5 and 8 (N95): Mask D.

### Procedure

In this experimental design, we measured the leakage rate using various masks in sequence from 0 to 8. The validation discussion of the fit tester for OPC and CNC is still ongoing. Therefore, this study was conducted for the same pairs of masks, specifically Measure 1 and Measure 6, Measure 2 and Measure 7, and Measure 3 and Measure 8, to confirm the consistency between the following methods.

The ILRs were measured using the following six mask-wearing options (Measures 0–5), and the FF was measured using three options (Measures 6–8) as follows:

• *Measure 0* (Initial): Mask A, the mask that the individual was wearing when recruited (including a variety of mask types).
• *Measure 1* (Surgical): Mask B, worn with the pleats spread out and the filter wire on the nose pressed down.
• *Measure 2* (Surgical-W): Mask B with the nose-fit wire adjusted to a W shape prior to wearing, according to the shape of the participant’s nose. Different ways of bending affect the fit, so the same researchers bent the wires on all the masks to standardize the procedure.
• *Measure 3* (S-S double): A double mask with two layers of Mask B; after quantifying Measure 2, we added an additional Mask B over the first mask.
• *Measure 4* (S-U double): Mask C worn over Mask B (double mask). After Measure 3, Mask B (the top layer) was removed, and Mask C was added.
• *Measure 5* (N95): Mask D, the researchers taught and helped the participants to put on masks to ensure that there were no gaps between the face and the respirator.
  Thereafter, a standard fit test was performed on a subgroup of participants (n = 39) for validation.
• *Measure 6* (Surgical): Mask B, using the same wearing method as for Measure 1.
• *Measure 7* (Surgical-W): Mask B, using the same wearing method as for Measure 2.
• *Measure 8* (N95): Mask D, using the same wearing method as for Measure 5.

### Inward leakage rate during mask use (fit check)

A fit tester with an optical particle counter (MT-05U; SIBATA, Tokyo, Japan) was used to count the number of ambient aerosol particles outside and inside the mask (particle size >0.3 µm in diameter). The fit check involved inserting a probe between the face and mask, without damaging the mask.

The ILR was calculated using the following equation:

$$\begin{array}{rl} \mathrm{ILR} & =\text{number of particles entering the mask}\times 100\\ & \;/\text{number of airborne particles outside the mask}. \end{array}$$

### Fit factor for mask use (fit test)

To validate the fit check results, we also performed a formal fit test using an onboard CNC fit tester count ambient aerosols (ranging from 0.15 to 1 µm in size) (AccuFIT 9000®PRO Model 3000-J1, Kanomax Japan Inc., Osaka, Japan). Exercises from the 29 CFR 1910.134 US OSHA modified ambient aerosol CNC QNFT protocol [24] were used.

The overall FF was calculated using the mean of the individual exercise FFs and converted to the ILR based on the computed FPM number for comparison with results from the fit check.

Exercises from the 29 CFR 1910.134 US OSHA modified ambient aerosol CNC QNFT protocol [24] were used in the following order: (1) normal breathing, (2) bending over, (3) moving the head upward and downward, (4) turning the head from side to side, (5) talking (counting backward from 100), and with (6) normal breathing. We added normal breathing (1 and 6) before and after applying the OSHA protocol.

The FF was calculated using the following equation:

$$\begin{array}{rl} \mathrm{FF} & =\text{number of airborne particles outside the mask}\\ & \;/\text{number of particles that entered the mask} \end{array}$$

### Filter-collection efficiency and inhalation resistance

The filter-collection efficiency and inhalation resistance test were examined using NaCl (particle size: 0.075 ± 0.02 µm) at a flow rate of 85 L/min with a mask performance test apparatus (AFT model 8130A, TSI, Shoreview, MN, USA).

### Statistical analysis

Statistical power was calculated using the “MKpower” package for R (R Foundation for Statistical Computing, Vienna, Austria). Descriptive statistics were used to report the demographic characteristics. Multiple linear regression was used to assess the factors (age, sex, height, weight, presence of allergy, mask size, HCW / general population, and face size) affecting the leakage rate of each mask-wearing option. A linear mixed-model analysis was used to compare the different mask options adjusting for intra-individual correlations and potential confounders. Spearman’s correlation coefficient (ρ) was used to assess the correlation between the mask option and the face size parameters. The Wilcoxon signed-rank test with a Bonferroni correction was used for the paired comparisons between the mask options. Because the leakage rate ranged from 0% to 100% and was affected by the mask choice, some analyses were based on nonparametric methods. All the analyses were performed using SPSS version 25 (IBM Corp., Armonk, NY, USA). Statistical significance was set as *P* < 0.05.

## Results

Fifty-four participants (20 men and 34 women; 31 from the general population and 23 HCWs) were included in this study (Table 2). As the standard deviation of the difference in the leakage rate in this study was 25%, the sample size of 54 provided a power of 90.3% if the difference was greater than 15% for α = 0.05.

**Table 2:** Baseline background and demographic characteristics of the study participants (N = 54)

**Variables**	**Value**
**Sex, n (%)**	
Male	20 (37.0%)
Female	34 (63.0%)
**Age (years), mean ± SD**	36.4 ± 13.7
**HCW, n (%)**	23 (42.6%)
**Smoker, n (%)**	4 (7.4%)
**History of allergies, n (%)**	28 (51.9%)
**Height (cm), mean ± SD**	163.72 ± 8.60
**Weight (kg), mean ± SD**	58.42 ± 12.02
**Body mass index (kg/m^2^), mean ± SD**	21.68 ± 3.26
**Room temperature (°C), mean ± SD**	24.46 ± 1.54
**Room humidity (%), mean ± SD**	53.61 ± 1.76

HCW, healthcare worker; SD, standard deviation.

Room temperature and humidity were assessed at the site of testing.

Table 3 shows the relationship between the ILR and the size of each facial section for each mask option. NH was significantly correlated with ILR (ρ = 0.522, *P* < 0.05) with Measure 1 for the small mask. In the next experiment, Measure 2 with an adjusted nose-fit wire did not show a relationship between the NH and the ILR (ρ = 0.132; *P* = 0.58). When the nose-fit wire was adjusted, the relationship between the NH and the ILR was eliminated. A significant correlation between the ILR and the ear-to-nose distance, LW, and NH were found in Measure 5 (N95). No significant association was found between the ILR and the participant characteristics (face size, sex, occupation, weight, and height). A significant relationship between age and ILR is observed, with a slight increase of 0.08% ILR for each year of age. (Table 4).

**Table 3:** Correlation between face size parameters and the inward leakage rate using different mask-wearing options

**Mask type, combination, and size**		**Face size parameter**				

		**Menton to sellion**	**Menton to glabella**	**Ear to nose**	**Labial width**	**Nose height**
**Measure 0^a^**						
Regular	ρ	0.085	−0.082	0.044	−0.027	0.156
	P value	0.639	0.65	0.803	0.879	0.378
	N	33	33	34	34	34
Small	ρ	0.006	0.238	0.525	0.181	**0.588**
	P value	0.986	0.509	0.119	0.593	**0.074**
	N	10	10	10	11	10

**Measure 1^b^**						
Regular	ρ	0.097	−0.014	0.179	−0.037	−0.01
	P value	0.604	0.939	0.335	0.845	0.956
	N	31	31	31	31	31
Small	ρ	0.202	0.034	0.343	0.181	**0.522**
	P value	0.406	0.891	0.139	0.432	**0.018**
	N	19	19	20	21	20

**Measure 2^c^**						
Regular	ρ	0.097	−0.014	0.179	−0.037	−0.01
	P value	0.604	0.939	0.335	0.845	0.956
	N	31	31	31	31	31
Small	ρ	−0.309	−0.49	0.087	0.074	0.132
	P value	0.198	**0.033**	0.714	0.749	0.58
	N	19	19	20	21	20

**Measure 3^d^**						
Regular	ρ	0.041	−0.056	0.07	−0.251	−0.023
	P value	0.828	0.77	0.713	0.18	0.902
	N	30	30	30	30	30
Small	ρ	−0.153	−0.138	0.218	0.231	0.223
	P value	0.519	0.561	0.342	0.301	0.332
	N	20	20	21	22	21

**Measure 4^e^**						
Regular	ρ	0.307	−0.077	−0.034	−0.144	−0.141
	P value	0.099	0.686	0.86	0.447	0.458
	N	30	30	30	30	30
Small	ρ	−0.161	−0.358	0.156	0.278	0.149
	P value	0.498	0.121	0.5	0.21	0.518
	N	20	20	21	22	21

**Measure 5^f^**						
Regular	ρ	0.246	0.163	**0.376**	**0.335**	**0.36**
	P value	0.086	0.257	**0.007**	**0.015**	**0.009**
	N	50	50	51	52	51

ρ, Spearman’s correlation coefficient; ILR, inward leakage rate.

^a^Measure 0: Mask A, the mask that the participant was wearing when recruited (including a variety of mask types).

^b^Measure 1: Mask B (surgical mask), worn with the pleats spread out and the filter wire on the nose pressed down.

^c^Measure 2: Mask B (surgical mask) with the nose nose-fit wire adjusted to a W shape, according to the shape of the participant’s nose, prior to wearing.

^d^Measure 3: Two layers of Mask B (surgical mask).

^e^Measure 4: Mask C (urethane mask) worn over Mask B (surgical mask).

^f^Measure 5: Mask D (N95).

**Table 4:** Assessment of the factors affecting the inward leakage rate of each mask-wearing option.

**Measurement**	**Parameter**	**Mean ** **difference**	**P value**	**95% Confidence ** **Interval**	

				**Lower ** **Bound**	**Upper Bound**
**Measure 1** (Surgical)	Intercept	−37.62	0.766	−291.41	216.16
	Gender	−18.97	0.208	−48.93	10.99
	Age	−0.38	0.265	−1.05	0.30
	Height	0.65	0.419	−0.96	2.26
	Weight	0.24	0.604	−0.68	1.16
	Allergy	1.65	0.834	−14.11	17.40
	HCWs	−11.22	0.267	−31.37	8.93
	Nose height	1.05	0.163	−0.44	2.54
**Measure 2** (Surgical-W)	Intercept	124.84	0.387	−163.38	413.06
	Gender	−5.59	0.742	−39.61	28.44
	Age	−0.45	0.248	−1.21	0.32
	Height	−0.37	0.683	−2.20	1.45
	Weight	−0.13	0.798	−1.18	0.91
	Allergy	4.75	0.595	−13.14	22.64
	HCWs	−8.03	0.482	−30.92	14.85
	Nose height	0.76	0.368	−0.93	2.46
**Measure 3** (S-S double)	Intercept	−21.67	0.890	−336.83	293.49
	Gender	−16.45	0.377	−53.66	20.75
	Age	0.31	0.457	−0.53	1.15
	Height	0.18	0.853	−1.81	2.18
	Weight	0.25	0.662	−0.89	1.39
	Allergy	−7.94	0.417	−27.50	11.63
	HCWs	5.40	0.666	−19.63	30.42
	Nose height	0.63	0.499	−1.23	2.48
**Measure 4** (S-U double)	Intercept	−40.94	0.768	−319.70	237.81
	Gender	−14.47	0.380	−47.38	18.44
	Age	−0.46	0.216	−1.20	0.28
	Height	0.71	0.424	−1.06	2.47
	Weight	−0.18	0.727	−1.19	0.83
	Allergy	−4.23	0.624	−21.53	13.08
	HCWs	−5.71	0.605	−27.84	16.42
	Nose height	0.29	0.722	−1.35	1.93
**Measure 5** (N95)	Intercept	1.89	0.893	−26.30	30.07
	Gender	−0.84	0.615	−4.16	2.49
	Age	0.08	0.035	0.01	0.16
	Height	−0.04	0.626	−0.22	0.14
	Weight	0.03	0.594	−0.08	0.13
	Allergy	−0.68	0.440	−2.43	1.07
	HCWs	1.46	0.196	−0.78	3.69
	Nose height	0.11	0.203	−0.06	0.27

HCWs, health care workers.

*Multiple linear regression analysis

The Measure 5 ILR was compared with the Measure 1–4 ILRs using a linear mixed model by adjusting for potential confounding factors. No significant relationship was found between the ILR and the variables relating to the participant characteristics. Regarding mask-wearing options, Measures 1–4 differed significantly from Measure 5 (*P* < 0.001) for all parameters tested (Table 5).

**Table 5:** Linear mixed-model analysis comparing the different mask-wearing options, adjusted potential confounders

**Parameter**	**Difference ** **(ILR)**	**P value**	**95% Confidence ** **Interval**	
			**Lower Bound**	**Upper Bound**
**Intercept**	−36.04	0.687	−215.32	143.23
**Gender**	−11.21	0.283	−32.03	9.62
**Age**	−0.18	0.441	−0.65	0.29
**Allergy**	−1.27	0.816	−12.21	9.68
**Mask size**	−0.72	0.887	−10.64	9.21
**Height**	0.22	0.699	−0.91	1.34
**Weight**	0.03	0.934	−0.64	0.69
**HCWs**	−3.74	0.595	−17.81	10.34
**Nose height**	0.56	0.281	−0.48	1.60
**Measure 1 (Surgical)**	**82.34**	**<0.001**	73.79	90.90
**Measure 2 (Surgical-S)**	**54.19**	**<0.001**	45.64	62.75
**Measure 3 (S-S double)**	**40.43**	**<0.001**	31.79	49.08
**Measure 4 (S-U double)**	**42.85**	**<0.001**	34.21	51.50
**Measure 5 (N95)**	Reference	.	.	.

ILR, inward leak rate; HCWs, health care workers.

*Dependent variable: ILR.

Participant-specific differences and distribution of the median ILR for each measurement are shown in Table 6. The median ILR from all participants was 96.44% for the surgical mask (Measure 1); 50.82%, Measure 2; 38.66%, Measure 3; 37.21%, Measure 4. For Measure 5, 49 participants had an ILR <5%.

**Table 6:** The inward leakage rate as determined by the quantitative fit test with different mask-wearing options

	**Mask-wearing option**								

	**Measure 0 (Initial)**			**Measure 1 (Surgical)**			**Measure 2 (Surgical-W)**		

	**Median**	**25th**	**75th**	**Median**	**25th**	**75th**	**Median**	**25th**	**75th**
**All**	100.00	68.51	100.00	96.44	72.83	100.00	50.82	35.68	76.24
**HCWs**	93.78	74.46	100.00	93.62	65.32	100.00	58.01	40.67	75.81
**General population**	100.00	51.59	100.00	100.00	73.02	100.00	43.26	33.33	74.34
**Male**	100.00	69.59	100.00	86.28	68.65	100.00	39.78	31.35	59.81
**Female**	100.00	70.20	100.00	98.60	77.08	100.00	59.34	40.01	78.07

**ILR**	**n (%)**			**n (%)**			**n (%)**		
**100%**	25 (46.3)			23 (42.6)			4 (7.4)		
**50–99.9%**	13 (24.1)			26 (48.1)			24 (44.4)		
**5–49.9%**	9 (16.7)			5 (9.3)			26 (48.1)		
**<5%**	0 (0)			0 (0)			0 (0)		

	**Mask-wearing option**								

	**Measure 3 (S-S double)**			**Measure 4 (S-U double)**			**Measure 5 (N95)**		

	**Median**	**25th**	**75th**	**Median**	**25th**	**75th**	**Median**	**25th**	**75th**
**All**	38.66	16.78	55.40	37.21	25.39	59.23	0.78	0.29	2.43
**HCWs**	41.05	18.51	53.58	40.00	23.69	62.87	0.58	0.30	1.17
**General population**	28.94	17.05	56.34	35.39	27.03	49.08	0.93	0.34	2.71
**Male**	34.46	18.50	51.88	33.48	24.57	52.96	0.96	0.48	2.28
**Female**	38.66	17.07	65.01	40.33	28.28	62.53	0.78	0.29	2.24

**ILR**	**n (%)**			**n (%)**			**n (%)**		
**100%**	4 (7.4)			3 (5.6)			0 (0)		
**50–99.9%**	13 (24.1)			14 (25.9)			0 (0)		
**5–49.9%**	36 (66.7)			37 (68.5)			5 (9.3)		
**<5%**	1 (1.9)			0 (0)			49 (90.7)		

	**Mask-wearing option**								

	**Measure 6 (Surgical)**			**Measure 7 (Surgical-W)**			**Measure 8 (N95)**		

	**Median**	**25th**	**75th**	**Median**	**25th**	**75th**	**Median**	**25th**	**75th**
**Total**	37.33	30.31	50.00	20.41	16.67	33.15	2.39	1.61	3.17
**HCWs**	48.10	33.33	50.00	27.66	16.69	33.33	2.11	1.59	2.76
**General population**	33.33	26.62	48.33	20.00	15.11	29.69	2.65	1.70	3.61
**Male**	37.33	27.09	50.00	27.83	15.89	33.33	2.90	2.20	3.64
**Female**	36.65	32.86	49.44	20.30	16.67	31.15	1.96	1.50	2.71

**ILR**	**n (%)**			**n (%)**			**n (%)**		
**100%**	0 (0)			0 (0)			0 (0)		
**50–99.9%**	12 (30.8)			1 (2.6)			0 (0)		
**5–49.9%**	27 (69.2)			36 (94.7)			4 (10.5)		
**<5%**	0 (0)			1 (2.6)			34 (89.5)		

HCW, healthcare worker; ILR, inward leakage rate.

**Fig. 2:**
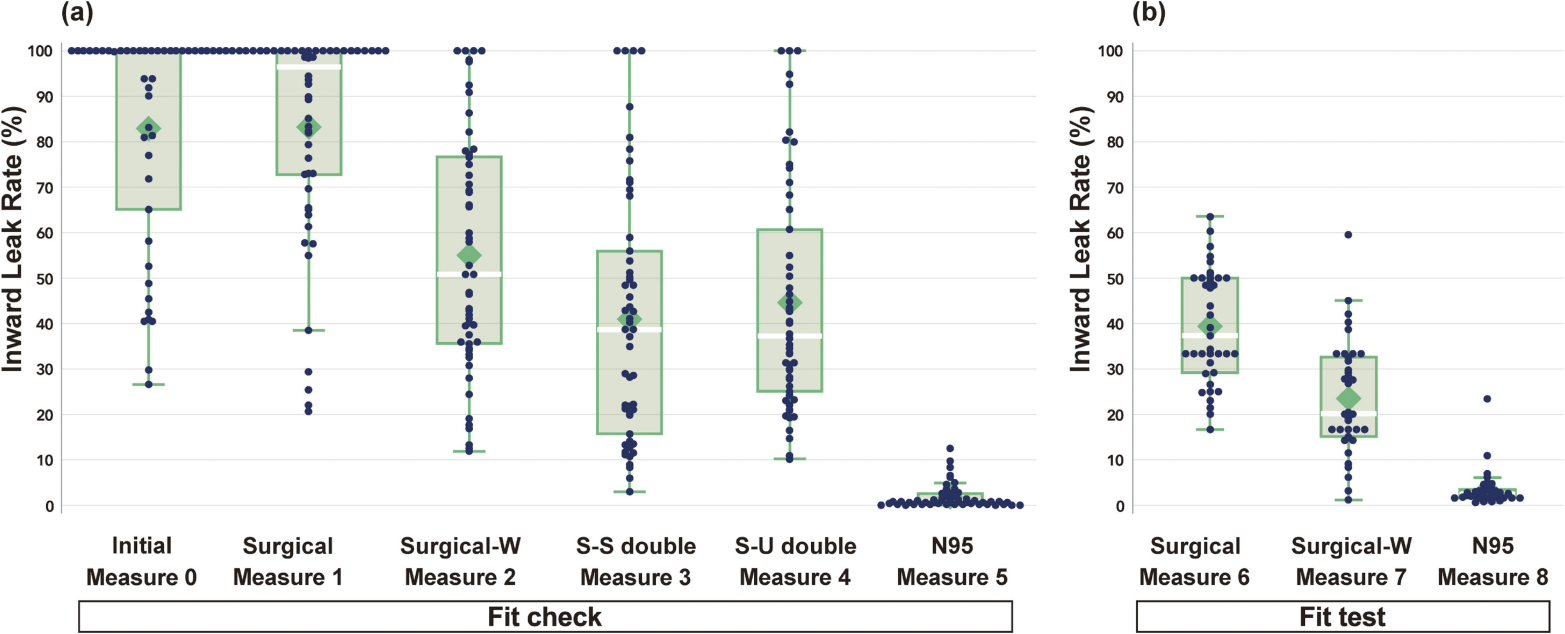
Box plot of the inward leakage rates for the different mask-wearing options Each dot indicates an individual leakage rate. (a) Fit check inward leakage rate (ILR) results for Measures 0–5, (b) Fit test ILR results for Measures 6–8

Figure 2 shows the ILR for each mask-wearing option. As shown in Table 1, participants in Measure 0 mainly wore surgical masks, and Measure 0 and Measure1 showed almost the same ILR trend. The ILR decreased significantly when utilizing the nose-fit wire was adjusted to improve the fit (Measure 2). In Measures 6–8, the results showed the same ILR trend when the ILR values were cross-checked with the results of the fit check (Measures 1, 2, and 5). N 95 masks (Measure 5, 8) reduced ILR more dramatically than any other mask.

In comparing the results of CNC fit tests and OPC fit checks, a consistent trend of ILR reduction was observed for Surgical (Measure 1 and 6) and Surgical-W (Measure 2 and 7). Conversely, the numerical values for leakage rates were lower in the CNC fit test. Similar results were obtained for N95 Masks (Measures 3 and 8).

The differences in ILRs for each mask-wearing option were compared (Table 7). The adjustment of the nose-fit wire of Mask B (Measure 2) reduced the ILR by an average of 28.57%, which was significantly lower than that measured without adjustment (*P* < 0.001). Measure 2 was found to be more effective than the unmodified surgical mask (Measure 1). For double masks, the ILR of Measure 3 was significantly lower than that of Measure 2 (*P* < 0.001). In case of double masking with a urethane mask on top (Measure 4), the ILR did not differ significantly from that of Measure 2. Additionally, the ILR did not differ significantly between Measures 3 and 4. Based on these results, the reduction in ILR for Measure 3 was not substantial; the upper surgical mask only slightly lowered the ILR by pressing down on the W-shaped nose-fit wire from above. The ILR of double masks did not differ significantly from single masks, and double masks were less effective than the N95 mask.

**Table 7:** Paired comparisons between mask-wearing options

**Pair**	**Median ILR**	**IQR**		**Median ILR**	**IQR**	***P* value**	**Bonferroni’s correction**
**Measure 2 - Measure 1**	50.82	(35.36–76.82)	vs.	96.44	(72–100)	<0.001	<0.001
**Measure 3 - Measure 1**	38.66	(15.34–56.65)	vs.	96.44	(72–100)	<0.001	<0.001
**Measure 4 - Measure 1**	37.21	(24.86–61.76)	vs.	96.44	(72–100)	<0.001	<0.001
**Measure 5 - Measure 1**	0.78	(0.29–2.55)	vs.	96.44	(72–100)	<0.0001	<0.001
**Measure 3 - Measure 2**	38.66	(15.34–56.65)	vs.	50.82	(35.36–76.82)	<0.001	<0.001
**Measure 4 - Measure 2**	37.21	(24.86–61.76)	vs.	50.82	(35.36–76.82)	0.003	0.030
**Measure 5 - Measure 2**	0.78	(0.29–2.55)	vs.	50.82	(35.36–76.82)	<0.001	<0.001
**Measure 4 - Measure 3**	37.21	(24.86–61.76)	vs.	38.66	(15.34–56.65)	0.106	1.000
**Measure 5 - Measure 3**	0.78	(0.29–2.55)	vs.	38.66	(15.34–56.65)	<0.001	<0.001
**Measure 5 - Measure 4**	0.78	(0.29–2.55)	vs.	37.21	(24.86–61.76)	<0.001	<0.001

*Wilcoxon signed rank test

Measure 1: Mask B (surgical mask), worn with the pleats spread out and the filter wire on the nose pressed down.

Measure 2: Mask B (surgical mask) with the nose nose-fit wire adjusted to a W shape, according to the shape of the participant’s nose, prior to wearing.

Measure 3: Two layers of Mask B (surgical mask).

Measure 4: Mask C (urethane mask) worn over Mask B (surgical mask).

Measure 5: Mask D (N95).

The performance of S-S double mask equaled that of the N95 mask filter, in terms of collection efficiency and inhalation resistance; however, the ILRs showed a significant difference (Table 1 and Table 6).

**Fig. 3:**
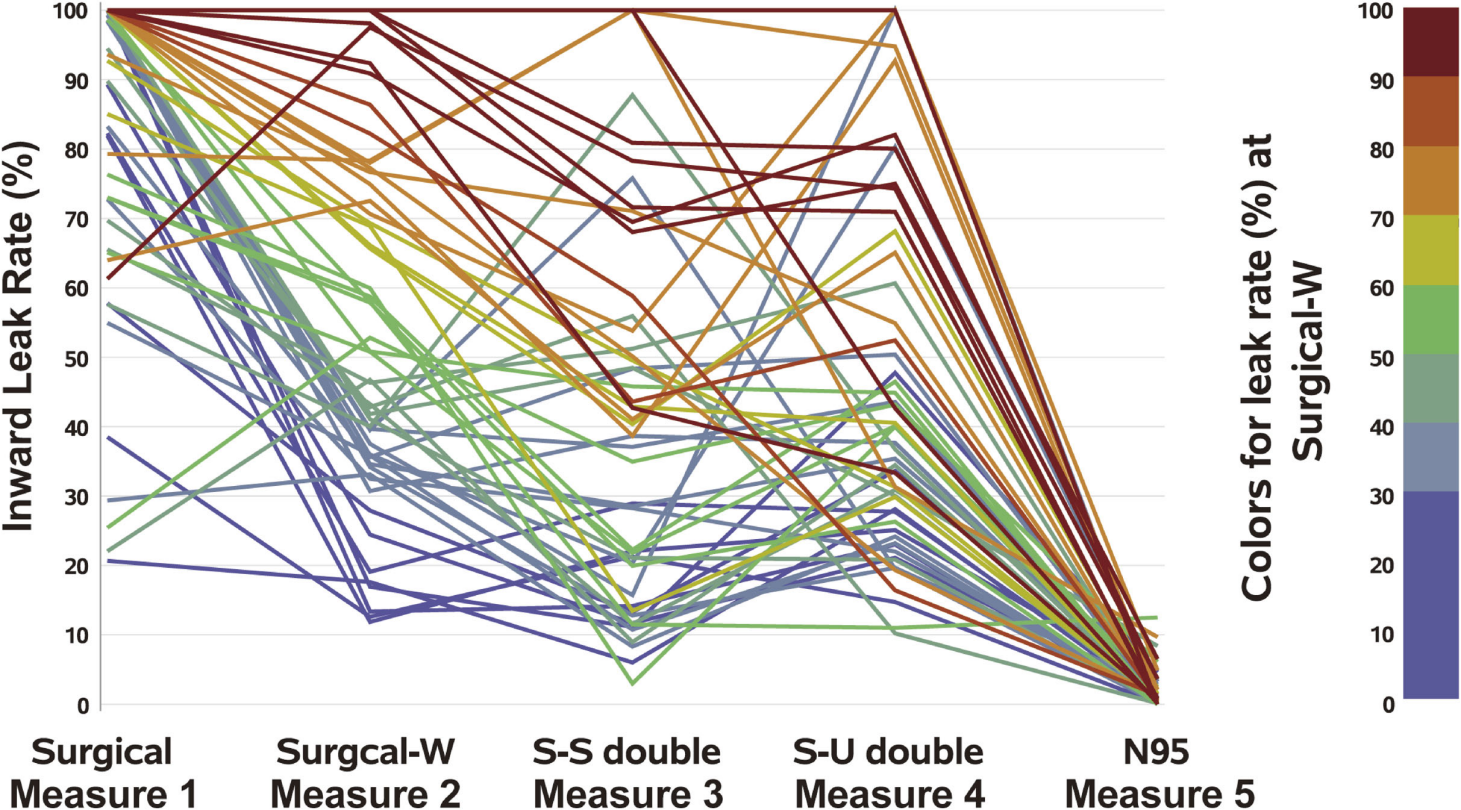
Change in the inward leakage rate according to the mask-wearing options The colors indicate the inward leakage rate for the Mask B (surgical mask) with the nose-fit wire bent in a W shape (red: high leakage, yellow: moderate leakage, blue: low leakage)

A decrease (improvement) and an increase (deterioration) were both seen in the ILRs of double masks. The reduction in the ILR with a double mask was not large; specifically, the ILRs were lower with N95 masks than with surgical masks in almost all participants. The reduction in the ILR was confirmed using N95 FFRs in 53 participants (Fig. 3).

## Discussion

In this study, we found that the mask-wearing option made a significant difference in fit, which in turn led to a difference in the number of microdroplets being transmitted. Additionally, modifying the mask-wearing technique did not show a consistent effect.

**Fig. 4:**
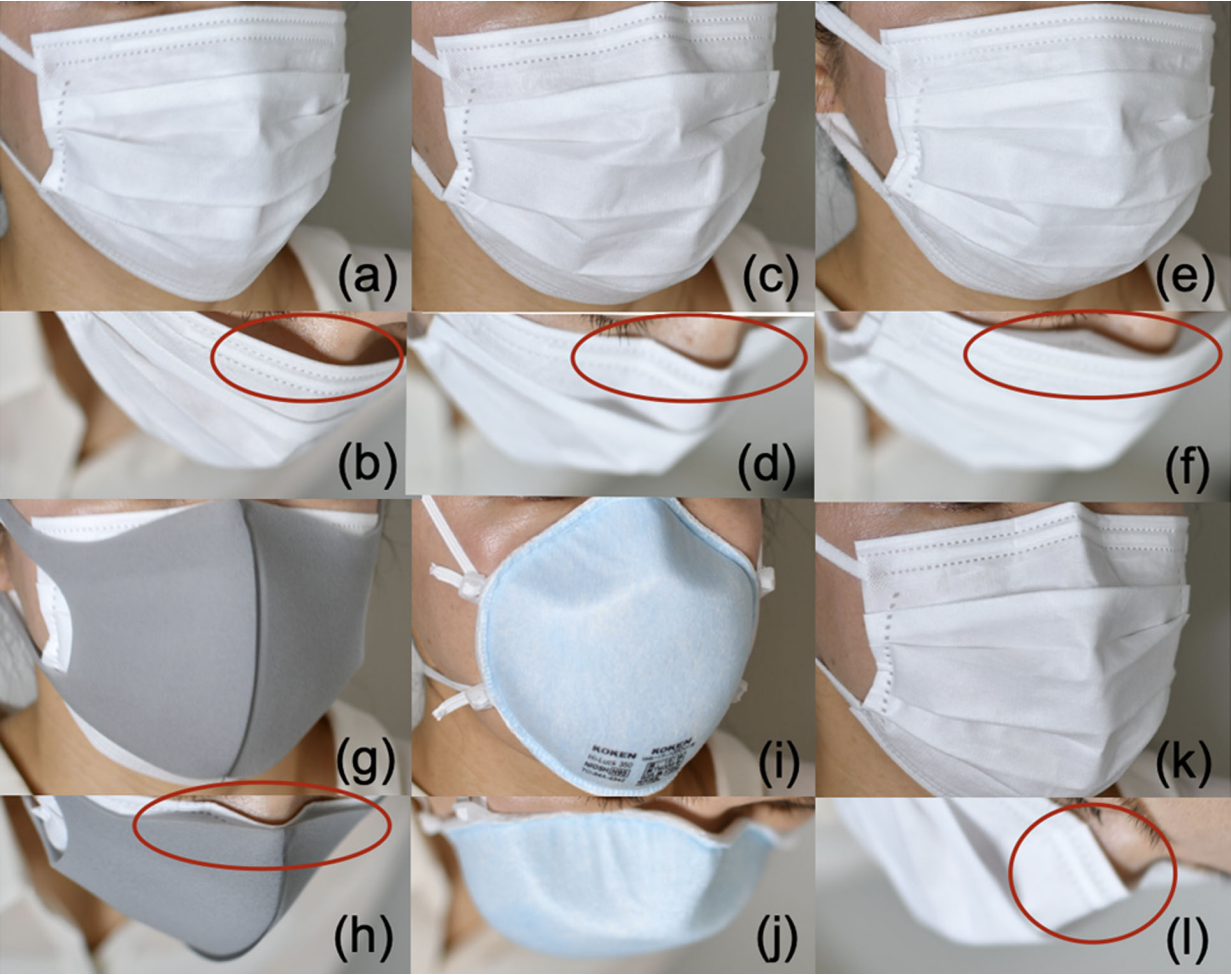
Wearing conditions of each mask and the gap between the nose section and the face (a, b) Measures 1 and 6 (Surgical mask). Mask B: The mask was adjusted by pressing down on the nose-fit wire after mask application. This left the wire “floating.” (c, d) Measures 2 and 7 (Surgical mask-W). Mask B with the nose-fit wire bent in a W shape. The nose-fit wire fits against the nose. (e, f) Measure 3 (S-S Double). Double surgical mask. After Measure 3, add Mask B. The nose-fit wire is attached to the nose. (g, h) Measure 4 (S-U Double). The inner surgical mask nose-fit wire is fitted to the nose. (i, j) Measures 5 and 8 (N95). The mask is worn after instructions or assistance; fits tightly between the face and mask. (k, l) The nose-fit wire completely should not be folded completely as this creates a gap between the mask and face.

Regarding the relationship between the size of each part of the face and the ILR, adjusting the nose-fit wire (Measure 2) eliminated the relationship between the NH and leakage (Table 3). Simply pressing the nose-fit wire to the shape of the nose was insufficient, because the nose-fit wire recovered its original shape (Fig. 4b). Additionally, if it was completely folded into a W shape, a gap formed (Fig. 4k and 4l). Based on observations made while handling the mask wire during the experiments, the ILR seemed to be influenced by the flexibility of the materials of the nose-fit wire.

The idea that a double mask is effective in preventing infection has often been discussed as it is expected to provide a double filtration effect. However, this opinion has been based solely on the filter and not on the sealed fit [27]. The enhancement in the efficacy of the double mask seemed to be primarily influenced by the pressing force of the overlaid mask. In cases wherein the fit was improved by the double mask, it was assumed that when an appropriate force was applied to the nose-fit wire of the outer mask, it effectively sealed the gap between the outer mask and the surgical mask with W shape below. Although the Centers for Disease Control and Prevention (CDC) predicted that double masking would be more effective than single masks, the ILR was lower with double masks, but the reduction was not statistically significant [18].

There are two reasons for the increase in the ILR due to double masking. The first is that the filter performance and inhalation resistance are enhanced by doubling the mask filter (53–102 Pa). Breath is less likely to pass through the filter and more likely to leak through the gap between the mask and face. The second reason is that, depending on the pressing force of the mask worn from above, the mask may be distorted, resulting in a more significant gap than when worn alone.

Measure 5 (N95) found that the ILR was associated with face size. Fixed-shape masks are not flexible and could sometimes be difficult to fit on the face. While N95 FFRs are manufactured to ensure filter performance, the proper selection of masks, education, and training may improve protection performance [28–31]. With a sufficiently low ILR and an absolute face fit, the N95 FFRs were significantly more effective than the surgical masks.

The fit and leakage rate of masks is assumed to be influenced by the subjects’ skeletal structure. However, in this study, the impact of the subject’s characteristics on ILR was insignificant. While there was a statistically significant correlation between increasing age and ILR, the rate of leakage increase was minimal, and the effect was marginal, possibly occurring coincidentally. Further research with more subjects representing a diverse range of facial variations would be necessary to elucidate this point.

In the CNC fit test for surgical masks, the leakage rate values tended to overrate the mask’s performance excessively as in the previous report [25, 26]. The following two concerns have been raised regarding the use of the CNC-equipped fit testers in surgical masks. First, in the CNC fit test, the measured particle size was small, so the collection efficiency of the filter against the effects of Brownian motion, diffusion, and electrostatic force was enhanced, and the FF may have been better than the actual result. Second, unlike the N95 FFR, which has superior retention, the weight of the tube connected to the jig made the mask prone to distortion and the assembly broke down easily when the face moved. This made exact measurements difficult and may not be useful when determining the actual surgical mask FF. Japan does not have a user seal check policy, but for surgical masks, a quick fit check should be performed using the ILR.

This study has several limitations. First, some FPM may have passed through the mask filter and not entirely through the gap between the face and mask. Moreover, in the fit check, we assumed that the leakage was caused by insertion of a probe. However, because this study aimed to observe the change in the ILR, the comparison of ILR using the difference in the fit method was performed adequately. Second, the effectiveness of the mask depends on how the nose-fit wire is bent, and not all people can create a W-bend to the same standard. Third, this study used one type of commercially available mask. Thus, the effectiveness shown here may not be generalized across all corresponding mask types. However, we showed that it may be generalized in real-life common-use situations compared with Measure 0. Fourth, while the CDC experiment used a double mask with a cloth mask on top, this study used a urethane mask [18]. Other combinations may produce different results. Future studies involving more participants and different mask-wearing options are needed, although the interpretation is unlikely to change from this study.

A previous study has suggested that surgical masks can capture the influenza virus in large droplets [32]. Even if a mask is not 100% effective against aerosol droplets, infection may still be avoided due to a partial protective effect. Hence, a mask should be worn, as the potential to avoid infection depends on the amount of the virus inhaled. The advantages and disadvantages of wearing masks are being deliberated, and it is difficult to make uniform recommendations and to educate people on how to use masks [33, 34].

The use of masks along with other infection-control measures (such as physical distancing, avoiding crowded and poorly ventilated indoor spaces, and improving hand hygiene) must be implemented correctly to prevent infections [35, 36]. Numerous studies have reported that universal masking prevents the spread of droplets and reduces the number of infected people [37]. Masks are more effective at preventing droplet spread than at preventing inward penetration. As shown in this study, it is difficult to prevent most aerosol droplets from entering the surgical mask, and the use of the N95 FFR or PAPR is recommended for this purpose [38]. In a pandemic, it is desirable to increase the availability of better fitting masks and to be able to operate the economy while wearing masks consistently. However, social issues such as the stress of constant mask-wearing, shortage of high-quality masks, and competition for masks must also be considered. The scientific evidence from this study may contribute to solving these problems.

## Conclusions

The study findings suggest that N95 masks alone can demonstrate effectiveness in many cases. On the other hand, maximizing the functionality of surgical masks depends on their proper usage. The modification of masks does not necessarily guarantee consistent effectiveness. These results highlight that wearing a properly selected, sealed mask can overcome simultaneous inward and outward leakage, emphasizing the crucial role of a “good fit” in enhancing overall mask efficiency. Nevertheless, mask-wearing may lead to unexpected infection outcomes without evidence from quantitative investigations. Therefore, ongoing innovative efforts are essential to enhance the fit and performance of surgical masks. Providing education grounded in explanations using quantitative experimental data is essential to prevent adverse outcomes.

## Abbreviations

ASTM: American Society for Testing and Materials
CDC: US Centers for Disease Control and Prevention
CNC: condensation nucleus counter
COVID-19: coronavirus disease
FF: fit factor
FFR: filtering facepiece respirator
FPM: fine particulate matter
HCW: healthcare worker
ILR: inward leakage rate
LW: labial width
MSL: menton-sellion length
NH: nose height
NIOSH: US National Institute for Occupational Safety and Health
OSHA: US Occupational Safety and Health Administration
PAPR: powered air-purifying respirators
PFE: particle filtration efficiency
QNFT: quantitative fit testing
SARS-CoV-2: severe acute respiratory syndrome coronavirus 2

## Declarations

### Availability of data and materials

The datasets that were used and analyzed in the current study are available from the corresponding author upon reasonable request.

### Author’s information

**ORCID**

Kazunari Onishi http://orcid.org/0000-0001-5791-4418
