# Correction to: Comparison of the inward leakage rate between N95 filtering facepiece respirators and modified surgical masks during the COVID-19 pandemic

**DOI:** 10.1265/ehpm.24-00196

**Published:** 2024-08-01

**Authors:** Kazunari Onishi, Masanori Nojima

**Affiliations:** 1 Division of Environmental Health, Graduate School of Public Health, St. Luke’s International University, 3-6-2 Tsukiji Chuo-ku, Tokyo 104-0045, Japan; 2 Center for Translational Research, The Institute of Medical Science Hospital, The University of Tokyo, 4-6-1 Shirokanedai, Minato-ku, Tokyo 108-8639, Japan

**Correction to: *Environ Health Prev Med* 29, 8 (2024)**

**https://doi.org/10.1265/ehpm.23-00303**

After this article was published [1], it was discovered that Table 6 contained errors due to incorrect transcription, with the values for Measure 2, 3, 4, and 7 inaccurately written. The numerical data in the Abstract Graphic, derived from the erroneous Table 6, also contained inaccuracies. Therefore, we will correct it as follows.

**Table 6:** The inward leakage rate as determined by the quantitative fit test with different mask-wearing options

	**Mask-wearing option**								

	**Measure 0 (Initial)**			**Measure 1 (Surgical)**			**Measure 2 (Surgical-W)**		

	**Median**	**25th**	**75th**	**Median**	**25th**	**75th**	**Median**	**25th**	**75th**
**All**	100.00	68.51	100.00	96.44	72.83	100.00	50.82	35.68	76.24
**HCWs**	93.78	74.46	100.00	93.62	65.32	100.00	58.01	40.67	75.81
**General population**	100.00	51.59	100.00	100.00	73.02	100.00	43.26	33.33	74.34
**Male**	100.00	69.59	100.00	86.28	68.65	100.00	39.78	31.35	59.81
**Female**	100.00	70.20	100.00	98.60	77.08	100.00	59.34	40.01	78.07

**ILR**	**n (%)**			**n (%)**			**n (%)**		
**100%**	25 (46.3)			23 (42.6)			4 (7.4)		
**50–99.9%**	13 (24.1)			26 (48.1)			24 (44.4)		
**5–49.9%**	9 (16.7)			5 (9.3)			26 (48.1)		
**<5%**	0 (0)			0 (0)			0 (0)		

	**Mask-wearing option**								

	**Measure 3 (S-S double)**			**Measure 4 (S-U double)**			**Measure 5 (N95)**		

	**Median**	**25th**	**75th**	**Median**	**25th**	**75th**	**Median**	**25th**	**75th**
**All**	38.66	16.78	55.40	37.21	25.39	59.23	0.78	0.29	2.43
**HCWs**	41.05	18.51	53.58	40.00	23.69	62.87	0.58	0.30	1.17
**General population**	28.94	17.05	56.34	35.39	27.03	49.08	0.93	0.34	2.71
**Male**	34.46	18.50	51.88	33.48	24.57	52.96	0.96	0.48	2.28
**Female**	38.66	17.07	65.01	40.33	28.28	62.53	0.78	0.29	2.24

**ILR**	**n (%)**			**n (%)**			**n (%)**		
**100%**	4 (7.4)			3 (5.6)			0 (0)		
**50–99.9%**	13 (24.1)			14 (25.9)			0 (0)		
**5–49.9%**	36 (66.7)			37 (68.5)			5 (9.3)		
**<5%**	1 (1.9)			0 (0)			49 (90.7)		

	**Mask-wearing option**								

	**Measure 6 (Surgical)**			**Measure 7 (Surgical-W)**			**Measure 8 (N95)**		

	**Median**	**25th**	**75th**	**Median**	**25th**	**75th**	**Median**	**25th**	**75th**
**Total**	37.33	30.31	50.00	20.41	16.67	33.15	2.39	1.61	3.17
**HCWs**	48.10	33.33	50.00	27.66	16.69	33.33	2.11	1.59	2.76
**General population**	33.33	26.62	48.33	20.00	15.11	29.69	2.65	1.70	3.61
**Male**	37.33	27.09	50.00	27.83	15.89	33.33	2.90	2.20	3.64
**Female**	36.65	32.86	49.44	20.30	16.67	31.15	1.96	1.50	2.71

**ILR**	**n (%)**			**n (%)**			**n (%)**		
**100%**	0 (0)			0 (0)			0 (0)		
**50–99.9%**	12 (30.8)			1 (2.6)			0 (0)		
**5–49.9%**	27 (69.2)			36 (94.7)			4 (10.5)		
**<5%**	0 (0)			1 (2.6)			34 (89.5)		

HCW, healthcare worker; ILR, inward leakage rate.
